# Einsamkeit im Kindes- und Jugendalter. Zur Verbreitung eines Risikofaktors für die psychische Gesundheit unter 11- bis 15-jährigen deutschen Schülerinnen und Schülern

**DOI:** 10.1007/s00103-023-03728-x

**Published:** 2023-06-01

**Authors:** Raphael Schütz, Ludwig Bilz

**Affiliations:** Institut für Gesundheit, Fakultät für Soziale Arbeit, Gesundheit und Musik, Brandenburgische Technische Universität Cottbus-Senftenberg, Universitätsplatz 1, 01968 Senftenberg, Deutschland

**Keywords:** Einsamkeit, Kinder, Jugendliche, UCLA Loneliness Scale, HBSC, Loneliness, Children, Adolescence, UCLA Loneliness Scale, HBSC

## Abstract

**Hintergrund:**

Einsamkeit ist sowohl im öffentlichen als auch im wissenschaftlichen Diskurs ein zunehmend präsentes Thema. Es liegen inzwischen zahlreiche Forschungsergebnisse zur Verbreitung und zu den gesundheitsbezogenen Zusammenhängen von Einsamkeit bei Erwachsenen vor. Zur Einsamkeit im Kindes- und Jugendalter gibt es für Deutschland nur wenige Erkenntnisse. Vor diesem Hintergrund stellt dieser Beitrag Befunde zur Verbreitung von Einsamkeit bei 11- bis 15-jährigen deutschen Schüler*innen vor und untersucht Zusammenhänge mit soziodemographischen Merkmalen.

**Methoden:**

Die Studie „Health Behaviour in School-aged Children“ (HBSC) ist eine globale Forschungskooperation unter Schirmherrschaft der Weltgesundheitsorganisation (WHO). In Brandenburg erfasste die Studie Daten von 3819 Kindern und Jugendlichen an allgemeinbildenden Schulen in den Klassenstufen 5, 7 und 9 (*M*_*Alter*_ = 13,5, *SD* = 1,6). Einsamkeit wurde anhand der University of California, Los Angeles (UCLA) Loneliness Scale und eines Einzelitems erhoben.

**Ergebnisse:**

13,2 % der Befragten gaben an, sich „meistens“ einsam zu fühlen, „immer“ einsam fühlten sich 3,6 %. Einsamkeit war stärker ausgeprägt bei Mädchen, bei Schüler*innen mit der Geschlechtszuschreibung „divers“ sowie bei älteren Heranwachsenden und bei Kindern und Jugendlichen mit niedrigerem sozioökonomischen Status.

**Diskussion:**

Die Befunde weisen darauf hin, dass Einsamkeit bei Kindern und Jugendlichen ein weit verbreitetes Phänomen ist. Dass sich Mädchen, ältere Schüler*innen sowie Kinder und Jugendliche mit geringerem familiären Wohlstand einsamer fühlen, stimmt mit Ergebnissen aus anderen Ländern überein und liefert Ansatzpunkte für die Planung von Präventionsmaßnahmen. Es bedarf in Deutschland weiterer Forschung zu möglichen gesundheitsbezogenen Zusammenhängen von Einsamkeit.

## Abstract (en)

**Background:**

Loneliness is an increasingly present topic in both public and scientific discourse. There is increasing research on the prevalence of loneliness and health-related correlates in adults. However, there are only a few findings on loneliness in childhood and adolescence in Germany. Therefore, this study aims to present findings on the prevalence of loneliness among 11- to 15-year-old German students and examines associations with socio-demographic characteristics.

**Methods:**

The Health Behaviour in School-aged Children (HBSC) study is a global research collaboration under the auspices of the World Health Organization (WHO). In the federal state of Brandenburg, Germany, the study collected data from 3819 children and adolescents in grades 5, 7, and 9 (*M*_age_ = 13.5,* SD* = 1.6) at schools for general education. Loneliness was assessed using the University of California, Los Angeles (UCLA) Loneliness Scale and a single item.

**Results:**

Of the respondents, 13.2% reported feeling lonely most of the time and 3.6% always. Loneliness was more prevalent among girls and non-binary students, as well as among older students and children and adolescents of lower socioeconomic status.

**Discussion:**

The findings indicate that loneliness is a widespread phenomenon among children and adolescents. The result that girls, older pupils and children, and adolescents with lower family wealth report feeling lonelier is consistent with findings from other countries and provides starting points for planning prevention measures. Further research is needed in Germany on possible health-related correlates of loneliness.

## Hintergrund

Das Thema Einsamkeit ist für die öffentliche Gesundheit und den politischen Diskurs von zunehmender Bedeutung [1]. Insbesondere durch die Corona-Pandemie erhielt es eine stärkere Aufmerksamkeit und Relevanz [2]. So wurde beispielsweise ein weiterer Anstieg der Einsamkeitsproblematik bei Jugendlichen erwartet [3].

In der Forschung wird Einsamkeit weitestgehend übereinstimmend in Anlehnung an Perlman und Peplau [4] definiert. Demnach ist Einsamkeit eine aversive Erfahrung aufgrund von quantitativ oder qualitativ mangelhaften sozialen Beziehungen. Es handelt sich dabei um eine subjektiv wahrgenommene und als leidvoll erlebte Diskrepanz zwischen erwünschten und vorhandenen Beziehungen [4, 5]. Das bedeutet einerseits, dass keine objektive soziale Isolation vorliegen muss, um Einsamkeit zu empfinden, und andererseits, dass Menschen auch alleine sein können, ohne sich einsam zu fühlen [4]. Eine weitere zentrale Differenzierung in emotionale und soziale Einsamkeit nimmt Weiss [6] vor: *Soziale Einsamkeit* bezieht sich auf einen Mangel an sozialen Netzwerken (z. B. im Kollegium, der Nachbarschaft und der Peergruppe), *emotionale Einsamkeit* meint ein Defizit oder einen Verlust an intimen Bindungen (z. B. romantische Partner- und enge Freundschaften). Wir definieren Einsamkeit in unserer Arbeit folglich in Anlehnung an Perlman und Peplau und an Weiss als ein aversives Gefühl, das aufgrund eines subjektiv erlebten Mangels an emotionalen und/oder sozialen Beziehungen entsteht.

Einsamkeit wird häufig als ein Phänomen des Alters betrachtet [7]. Sie ist jedoch auch in der Adoleszenz präsent [8] und hat bei Kindern und Jugendlichen über die letzten Jahre zugenommen [9, 10]. Einsamkeit bei jungen Menschen wird trotz hoher Prävalenz weniger intensiv erforscht als bei Älteren [11]. Dabei sind Jugendliche aufgrund der vielen physischen und psychischen Veränderungen sowie der sich wandelnden sozialen Beziehungen besonders vulnerabel für das Phänomen [12]. Sich in zwischenmenschlichen Beziehungen eingebunden zu fühlen, gilt als menschliches Grundbedürfnis, dessen Nichterfüllung als leidvoll erlebt werden kann [13].

So gehen bereits jüngere Kinder erste Freundschaften ein, die zunächst insbesondere durch gemeinsame Aktivitäten gekennzeichnet sind. Über den weiteren Verlauf der Kindheit und Jugend gewinnt dann die Qualität der Freundschaften und damit das Bedürfnis nach gegenseitigem Verständnis, Bestätigung und Mitgefühl an Bedeutung [14]. Entsprechend spielt für die Entstehung von Einsamkeit in der früheren Kindheit eher die Quantität und in der späten Kindheit und Jugend eher die Qualität von Freundschaften eine Rolle [14]. Auch die Akzeptanz von der Peergruppe wird in der Jugend zunehmend wichtig und ihr Fehlen kann eine weitere Quelle der Einsamkeit darstellen, während dies in der frühen Kindheit weniger ausschlaggebend ist [14]. Die Peergruppe nimmt im Zuge der schrittweisen Ablösung von den Eltern die Position der zentralen Bezugsgruppe ein [15]. Scheitern Jugendliche an dieser Entwicklungsaufgabe, kann dies zu Einsamkeit führen, da die Zeit mit der Familie im Jugendalter als soziale Isolation bewertet werden kann [12]. In der späteren Jugend entwickelt sich zudem das Bedürfnis nach romantischen Beziehungen [14]. Wird es nicht befriedigt, kann auch im Kontakt zur Peergruppe mangelnde Eingebundenheit empfunden werden [12]. Im Jugendalter kann Einsamkeit demnach sowohl mit nicht erfüllten Wünschen nach romantischen Beziehungen [14], fehlender Akzeptanz in der Peergruppe, mangelnden engen Freundschaften als auch, wie in der Kindheit, mit Viktimisierungserfahrungen, quantitativ fehlenden Freundschaften [16] und fehlender Unterstützung von Klassenkamerad*innen [17] assoziiert sein.

Einige dieser mit Einsamkeit assoziierten Faktoren lassen sich auch in den etablierten Ursachentheorien von Einsamkeit wiederfinden. Demnach besagt der *soziale Bedürfnisse-Ansatz*, dass sich Einsamkeit aufgrund einer mangelnden Befriedigung der individuellen Bedürfnisse nach sozialen oder emotionalen Beziehungen entwickelt [18, 19]. Der *kognitive Ansatz* geht davon aus, dass Einsamkeit dann entsteht, wenn eine Diskrepanz zwischen den vorhandenen und den subjektiv gewünschten sozialen Beziehungen existiert [4, 18]. Der *evolutionsbiologische Ansatz* postuliert, dass Einsamkeit als aversiv erlebtes Warnsignal vor dem Alleinsein fungiert. Evolutionär erfüllt es damit den Zweck, dass Menschen sich einer Gemeinschaft anschließen und somit die Weitergabe der eigenen Gene ermöglichen [20, 21].

Neben potenziellen Ursachen werden auch mögliche Folgen von Einsamkeit in der Forschung diskutiert [22]. Wobei sich bei Querschnittdesigns nicht eindeutig ableiten lässt, ob es sich um Ursachen oder Folgen von Einsamkeit handelt [23]. Mögliche Folgen von Einsamkeit sind vielfältig und reichen von wirtschaftlichen Konsequenzen [24] bis hin zu gesundheitlichen Beeinträchtigungen [25]. So steht Einsamkeit bei Kindern und Jugendlichen sowohl mit psychosomatischen Beeinträchtigungen wie körperlichen Schmerzen [9, 26–28] als auch mit psychischen Beschwerden wie Depressionen [29, 30], Ängsten [31, 32], geringerem Selbstwert und Wohlbefinden [33], Suizidalität [31, 34], Schlafproblemen [9, 26] sowie mit schlechteren schulischen Leistungen und einer größeren Schulunzufriedenheit [28] in Zusammenhang. Bei Erwachsenen erhöht Einsamkeit ferner das Risiko für körperliche Schmerzen [35], Herz-Kreislauf-Erkrankungen [36] und Mortalität [37, 38].

Die Angaben zur Verbreitung von Einsamkeit variieren je nach Land und Befragungsform. Im Rahmen der internationalen Studie „Health Behaviour in School-aged Children“ (HBSC) wurde Einsamkeit bei 11- bis 15-Jährigen in verschiedenen Ländern mit jeweils einem Item erfasst (z. B. in Dänemark mit der Frage „Fühlst du dich einsam?“ [10] und im Vereinigten Königreich mit der Frage „Wenn du an die letzte Woche denkst, hast du dich einsam gefühlt?“ [28]). Hierbei wurde als einsam gewertet, wer die oberen 2 Antwortkategorien gewählt hatte, z. B. „oft“ und „sehr oft“ [10] oder „sehr oft“ und „immer“ [28]. Die Verbreitung von Einsamkeit lag dabei je nach Land bei 6,3 % (Dänemark; [10]), 8,2 % (Vereinigtes Königreich; [28]) und 15 % (Finnland; [9]). Metaanalysen [39] und länderübergreifende Studien [40, 41] mit Kindern und Jugendlichen berichten eine Prävalenz zwischen 9,2 % und 14,4 % [39–41].

Für Deutschland liegen nur wenige repräsentative Studien mit Prozentangabe zur Prävalenz von Einsamkeit bei Kindern und Jugendlichen vor. Einige der deutschen Studien erfassen Einsamkeit erst ab 17 [42] oder 14 Jahren [43] mithilfe der UCLA Loneliness Scale (UCLA-Skala), sie proklamieren eine Verbreitung zwischen 14,2 % [42] und 26 % [43]. Die deutsche COPSY-Studie (Corona und Psyche; [44]) erfasst Einsamkeit auch in früheren Phasen der Kindheit und Jugend mit einem Item des Kidscreen-10. Hier werden die 11- bis 17-Jährigen gefragt, inwieweit sie sich in der letzten Woche einsam gefühlt haben. Dies traf auf 10 % der Befragten oft und auf 24 % manchmal zu. Die Studie wurde jedoch während der ersten Coronawelle und unter Lockdown-Bedingungen durchgeführt.

Die Erfassung von Einsamkeit mit nur einem Item gilt zwar als reliabel und valide [45], es wird jedoch diskutiert, ob die Selbstzuschreibung als „einsam“ aufgrund ihrer sozialen Stigmatisierung zu einer Unterschätzung der Verbreitung von Einsamkeit führen kann [26]. Ein indirektes Erhebungsinstrument wie die UCLA-Skala kann Einsamkeit möglicherweise akkurater erfassen [26], weshalb eine Kombination aus mehreren Erhebungsmethoden empfohlen wird [45]. Eccles et al. [26] berücksichtigen diesen Aspekt und verwenden in ihrer repräsentativen dänischen Studie neben einem Einzelitem auch die 4‑Item-UCLA-Skala von Roberts et al. [46]. Unter Verwendung des oberen Quartils der Antwortskala als Cut-off-Wert für Einsamkeit galten bei der Messung mit dem Einzelitem 7,7 % und bei der UCLA-Skala 14,2 % der Kinder und Jugendlichen als einsam [26]. Insgesamt sind die verschiedenen Studienergebnisse zur Verbreitung von Einsamkeit nur begrenzt miteinander vergleichbar. Neben unterschiedlichen Stichproben und Erhebungsmethoden (z. B. Einzelitem vs. Skala) variieren auch die verwendeten Cut-off-Werte.

Neben den methodischen Aspekten unterscheidet sich die Verbreitung von Einsamkeit auch in Abhängigkeit von soziodemographischen Merkmalen. Studien zu früheren HBSC-Erhebungen aus verschiedenen Ländern berichten Ergebnisse, die zwischen einem Anstieg der Einsamkeitsprävalenz mit zunehmendem Alter [9, 27, 28], keinen Altersunterschieden [10] und geschlechtsspezifischen Altersverläufen [26] divergieren. Hinsichtlich der Ausprägung nach Geschlecht wurde in vergangenen Erhebungen tendenziell eine höhere Ausprägung bei Mädchen ermittelt [9, 10, 26–28]. Als mögliche Ursachen werden z. B. eine unterschiedliche Sozialisierung [27] und die Verwendung von Einzelitem-Messungen diskutiert, verbunden mit einer höheren Bereitschaft von Mädchen, sich selbst als einsam zu beschreiben [26]. Bezüglich des sozioökonomischen Status erwiesen sich Heranwachsende aus Familien mit geringerem sozioökonomischen Status als einsamer [10, 28]. Zu Schulformunterschieden liegen unseres Wissens aktuell keine aussagekräftigen Befunde vor.

Zusammenfassend zeigt sich, dass Einsamkeit bei jungen Menschen in der Forschung vergleichsweise wenig Beachtung findet und insbesondere zur Verbreitung dieses Phänomens unter deutschen Kindern und Jugendlichen mehr aussagekräftige Daten erforderlich sind.

Ziel der vorliegenden Studie ist es demnach, zur Schließung dieser Forschungslücke beizutragen und die Verbreitung von Einsamkeit bei Kindern und Jugendlichen mithilfe einer repräsentativen deutschen Stichprobe zu erfassen. Hierbei soll sowohl anhand eines Einzelitems die Selbstzuschreibung der Kinder und Jugendlichen als „einsam“ sowie die Verbreitung von häufigen Einsamkeitsgefühlen mithilfe eines standardisierten Erhebungsinstruments ermittelt werden. Weiterhin sollen Zusammenhänge mit soziodemographischen Hintergrundbedingungen (Geschlecht, Alter, sozioökonomischer Status und Schulform) analysiert werden, um Ansatzpunkte für die weitere Forschung sowie für Präventions- und Interventionsmaßnahmen ableiten zu können.

Im Einzelnen werden folgende* Forschungsfragen *untersucht:

### Forschungsfrage 1.

Wie verbreitet ist Einsamkeit bei Kindern und Jugendlichen?

### Forschungsfrage 2.

Wie unterscheidet sich die Verbreitung von Einsamkeit je nach Altersgruppe, Geschlecht, Schulform und sozioökonomischem Status?

## Methoden

### Studiendesign und Stichprobe

Die Forschungsfragen werden anhand der Daten der HBSC-Studie Brandenburg 2022 untersucht. Die Daten wurden von Schüler*innen der Klassenstufen 5, 7 und 9 (Alter ca. 11,5; 13,5 und 15,5 Jahre) erhoben. Die Befragung wurde an zufällig ausgewählten allgemeinbildenden Schulen des Landes Brandenburg durchgeführt. Die Ziehung der Schulen erfolgte gemäß dem Forschungsprotokoll des HBSC-Konsortiums [47] mit dem Schichtungsmerkmal Schulform und unter Berücksichtigung der Schulgröße („probability-proportional-to-size sampling“, PPS).

Insgesamt wurden 365 Schulen kontaktiert, von denen 87 an der Befragung teilgenommen haben. Das entspricht einer Rücklaufquote von 23,8 % auf Schulebene. Insgesamt haben 4029 Schüler*innen die Befragung durchgeführt, was eine Rücklaufquote von 61,7 % auf Schüler*innenebene ergibt. Der finale Datensatz enthält Daten von 3819 Kindern und Jugendlichen (52,6 % Mädchen, 45,6 % Jungen, 1,8 % mit der Geschlechtszuschreibung „divers“). Das Durchschnittsalter beträgt 13,5 Jahre (*SD* = 1,6).

### Prozedere

Vor der Durchführung der HBSC-Studie erfolgten eine Genehmigung durch das Ministerium für Bildung, Jugend und Sport (MBJS) des Landes Brandenburg und eine Überprüfung der ethischen Unbedenklichkeit durch die Ethikkommission der Ärztekammer Hamburg. Die Schulleitungen konnten zwischen einer Online-Variante (Erhebung mittels Schulcomputern) und einer Tablet-Variante (mit Tablets des Forschungsteams) entscheiden. Dazu erhielten sie u. a. ein Einladungsschreiben (inkl. Anmeldebogen) und Informationsmaterialien zur Studie. Die Teilnahme an der HBSC-Studie war anonym und freiwillig. Es durften nur Schüler*innen teilnehmen, die eine unterschriebene Einverständniserklärung der Erziehungsberechtigten und (ab Klasse 7) eine selbst unterzeichnete Einverständniserklärung abgegeben hatten. Die Befragung erfolgte im Klassenverband von April bis Juli 2022 und dauerte etwa 45 min.

### Erhebungsinstrumente

*Einsamkeit* wurde mittels der 4‑Item-UCLA-Skala [46] erfasst. Die UCLA-Skala ist eines der meist verwendeten Instrumente, um Einsamkeit zu erheben [1], und wird als „Goldstandard“ der Einsamkeitsmessung bezeichnet [28]. Sie gilt als reliabel und valide [46] und wurde bereits in der dänischen HBSC-Studie erfolgreich bei Kindern und Jugendlichen angewendet [26]. In Deutschland wurde die 20-Item-Version der UCLA-Skala validiert [48]. Für die HBSC-Studie wurde die gekürzte 4‑Item-Variante von Muttersprachler*innen des HBSC-Teams in einem Rückübersetzungsverfahren ins Deutsche übertragen und in einem Pretest auf Verständlichkeit geprüft. Die Schüler*innen beurteilen 4 Aussagen dahingehend, wie oft sie sich in den letzten 12 Monaten entsprechend gefühlt haben (z. B. „Ich fühle mich von anderen isoliert“) mit den Antwortoptionen 0 = nie, 1 = selten, 2 = manchmal, 3 = meistens, 4 = immer. Somit kann der Summenscore maximal eine Ausprägung von 16 Punkten haben. Die interne Konsistenz der UCLA-Skala in der aktuellen Stichprobe ist sehr gut (α = 0,87).

Zusätzlich wird Einsamkeit mit einem Einzelitem aus dem Global Student Health Survey (GSHS; [40]) erfasst: „Wie oft hast du dich in den letzten 12 Monaten einsam gefühlt?“, mit den gleichen Antwortoptionen wie bei der UCLA-Skala. Es existieren keine konsensualen Cut-off-Werte für Einsamkeit [26]. Es wird jedoch davon ausgegangen, dass ein gewisses Maß an Einsamkeit unproblematisch ist [39], während hohe Ausmaße an Einsamkeit eher mit gesundheitlichen Beeinträchtigungen einhergehen [26, 28]. Daher wird der Cut-off-Wert für Einsamkeit in Anlehnung an andere Studien [9, 10, 28] zwischen die mittlere („manchmal“) und die beiden oberen („meistens“ und „immer“) Antwortkategorien gelegt. Das entspricht beim Einzelitem einem Cut-off-Wert von ≥ 3 und bei der UCLA-Skala von ≥ 9. So soll bei beiden Erhebungsinstrumenten zwischen gelegentlicher und häufiger Einsamkeit differenziert werden.

Das *Geschlecht* wurde anhand der Frage „Bist du ein Mädchen oder ein Junge?“ mit den Antwortoptionen „Junge“, „Mädchen“ und „divers“ erfasst. Das *Alter* wurde in Stufen entsprechend der Jahrgangsklassen abgebildet: 5. Klasse (ca. 11,5 Jahre), 7. Klasse (ca. 13,5 Jahre), 9. Klasse (ca. 15,5 Jahre). Die *Schulform* wurde im Rahmen der Erhebung mithilfe schulspezifischer Erhebungscodes automatisch erfasst.

Der familiäre Wohlstand, als Indikator für den *sozioökonomischen Status* der Schüler*innen, wurde mittels der *Family Affluence Scale *(FAS; [47]) erfasst. Die FAS setzt sich aus 6 Items zusammen, die das Vorliegen einzelner Wohlstandsgüter (z. B. „Besitzt deine Familie ein Auto?“ mit den Antwortoptionen „Nein“, „Ja, eins“ und „Ja, zwei oder mehr“) in der Familie des Heranwachsenden erfassen. In Anlehnung an das HBSC-Forschungsprotokoll [47] wurden die 6 Fragen zu einer Variable mit einem Score von 0–13 Punkten zusammengefasst und auf dieser Grundlage Quintile gebildet. Die unteren 20 % des Scores (Quintil 1) gelten als geringer, die mittleren 60 % (Quintil 2–4) als mittlerer und die oberen 20 % (Quintil 5) als hoher sozioökonomischer Status.

### Statistische Analyse

Zunächst erfolgen eine deskriptive Analyse und Prüfung bivariater Zusammenhänge mittels Kreuztabelle und Chi^2^‑Test. Anschließend wird zur multivariaten Absicherung der Zusammenhänge eine logistische Regression durchgeführt. Die Zusammenhangsmaße werden unter Angabe des Signifikanzniveaus (*p* < 0,05 bis *p* < 0,001) und der Effektstärke Cramers *V* (kleiner Effekt: *V* = 0,1, mittlerer Effekt: *V* = 0,3, großer Effekt: *V* = 0,5) angegeben.

## Ergebnisse

Die UCLA-Skala wurde von 3101 und das Einzelitem von 3284 Schüler*innen (44,5 % Jungen, 53,9 % Mädchen, 1,6 % mit der Geschlechtszuschreibung „divers“) beantwortet. Bei der UCLA-Skala ergab sich für die Kinder und Jugendlichen ein Mittelwert von 3,7 (*SD* = 3,6) und bei dem Einzelitem von 1,4 (*SD* = 1,1).

### Verbreitung von Einsamkeit

Von den befragten Kindern und Jugendlichen berichteten 27,6 % sich nie, 29,7 % sich selten, 25,9 % sich manchmal, 13,2 % sich meistens und 3,6 % sich immer einsam zu fühlen (Einzelitem).

Auf Grundlage der gewählten Cut-off-Werte sind demnach zwischen 10,8 % (UCLA-Skala) und 16,8 % (Einzelitem) der Kinder und Jugendlichen als einsam zu klassifizieren. Die Ergebnisse der beiden Erhebungsmethoden stehen in einem engen Zusammenhang (χ^2^ (1) = 757,28, *p* < 0,001, *V* = 0,495). Ein ausführlicher Überblick zur Verbreitung von Einsamkeit in den verschiedenen Subgruppen findet sich in Tab. 1.

**Table 1:** 

**UCLA-Skala**	***n***	**Einsamkeit in Prozent**	**Signifikanz**
*Gesamt*	3101	10,8	–
*Geschlecht*			χ^2^ (2) = 72,72, *p* < 0,001, *V* = 0,153
Mädchen	1674	13,7	
Jungen	1379	6,4	
Divers	48	35,4	
*Alterskategorie*			χ^2^ (2) = 35,21, *p* < 0,001, *V* = 0,107
5. Klasse	809	7,2	
7. Klasse	1241	9,3	
9. Klasse	1051	15,2	
*Schulform*			χ^2^ (4) = 8,90, *p* = 0,064, *V* = 0,054
Grundschule	674	8,0	
Oberschule	878	12,4	
Gymnasium	1403	11,0	
Gesamtschule	105	13,3	
Förderschule	38	7,9	
*Sozioökonomischer Status*			χ^2^ (2) = 16,94, *p* < 0,001, *V* = 0,075
Gering	469	16,0	
Mittel	2124	10,3	
Hoch	403	7,8	
**Einzelitem**	***n***	**Einsamkeit in Prozent**	**Signifikanz**
*Gesamt*	3284	16,8	–
*Geschlecht*			χ^2^ (2) = 130,56, *p* < 0,001, *V* = 0,199
Mädchen	1770	22,3	
Jungen	1461	9,1	
Divers	53	45,3	
*Alterskategorie*			χ^2^ (2) = 68,23, *p* < 0,001, *V* = 0,144
5. Klasse	886	10,3	
7. Klasse	1314	15,3	
9. Klasse	1084	23,9	
*Schulform*			χ^2^ (4) = 42,43, *p* < 0,001, *V* = 0,114
Grundschule	740	10,1	
Oberschule	921	22,1	
Gymnasium	1447	16,8	
Gesamtschule	128	16,4	
Förderschule	45	17,8	
*Sozioökonomischer Status*			χ^2^ (2) = 37,44, *p* < 0,001, *V* = 0,109
Gering	499	25,8	
Mittel	2237	16,0	
Hoch	424	11,8	

*UCLA* University of California, Los Angeles

### Zusammenhänge zwischen Einsamkeit und soziodemographischen Merkmalen

Laut der UCLA-Skala sind 6,4 % der Jungen, 13,7 % der Mädchen und 35,4 % der Befragten mit der Geschlechtszuschreibung „divers“ als einsam einzustufen. Beim Einzelitem gaben 9,1 % der Jungen, 22,3 % der Mädchen und 45,3 % der Befragten mit der Geschlechtszuschreibung „divers“ an, sich einsam zu fühlen. Dabei handelt es sich bei beiden Erhebungsinstrumenten um einen signifikanten Gruppenunterschied je nach *Geschlecht* (Tab. 1). Auch bezüglich der *Alterskategorien* zeigen sich signifikante Unterschiede in der Einsamkeit. Laut beider Erhebungsinstrumente fühlen sich Heranwachsende aus der 9. Klasse eher einsam als jüngere Schüler*innen. Bezüglich der *Schulformen *zeigt sich bei der Einsamkeitsprävalenz ein heterogenes Bild. Gemäß der UCLA-Skala ergibt sich insgesamt kein signifikanter Unterschied in der Verbreitung je nach Schulform, während dieser Zusammenhang mit dem Einzelitem vorliegt. Kinder und Jugendliche aus einem Zuhause mit geringerem *sozioökonomischen Status* waren gemäß den beiden Erhebungsinstrumenten signifikant einsamer als Heranwachsende mit mittlerem und hohem sozioökonomischen Status.

Um die Zusammenhänge zwischen Einsamkeit und den soziodemographischen Hintergrundmerkmalen multivariat abzusichern, werden nachfolgend die Ergebnisse einer logistischen Regression berichtet. Als Kriteriumsvariable dient einmal „Einsamkeit“ gemäß der UCLA-Skala und einmal gemäß dem Einzelitem. Sowohl für die UCLA-Skala (χ^2^ (10) = 113,21, *p* < 0,001) als auch für das Einzelitem (χ^2^ (10) = 223,22, *p* < 0,001) ist das binäre logistische Regressionsmodell signifikant. Die Regressionskoeffizienten und Odds Ratios (OR) für die einzelnen Prädiktoren werden in Tab. 2 berichtet.

**Table 2:** 

**UCLA-Skala**	***B***	***SE***	**OR**	**95** **%-KI**	**Signifikanz**
*Geschlecht*					
Jungen (Referenz)	–	–	–	–	–
Mädchen	0,80	0,13	2,23	1,72–2,90	***
Divers	1,95	0,33	7,02	3,68–13,39	***
*Alter*					
5. Klasse (Referenz)	–	–	–	–	–
7. Klasse	1,18	0,53	3,27	1,17–9,14	*
9. Klasse	1,68	0,52	5,39	1,94–14,97	***
*Schulform*					
Grundschule (Referenz)	–	–	–	–	–
Oberschule	−0,94	0,54	0,39	0,14–1,11	n. s.
Gymnasium	−1,04	0,55	0,35	0,12–1,03	n. s.
Gesamtschule	−0,64	0,60	0,53	0,16–1,72	n. s.
Förderschule	−1,61	0,90	0,20	0,03–1,15	n. s.
*Sozioökonomischer Status*					
Niedrig (Referenz)	–	–	–	–	–
Mittel	−0,50	0,15	0,61	0,45–0,82	***
Hoch	−0,75	0,23	0,48	0,30–0,75	*
**Einzelitem**	***B***	***SE***	**OR**	**95** **%-KI**	**Signifikanz**
*Geschlecht*					
Junge (Referenz)	–	–	–	–	–
Mädchen	1,05	0,11	2,87	2,31–3,57	***
Divers	1,97	0,30	7,14	3,97–12,85	***
*Alter*					
5. Klasse (Referenz)	–	–	–	–	–
7. Klasse	0,52	0,30	1,68	0,94–3,01	n. s.
9. Klasse	1,00	0,30	2,72	1,53–4,85	***
*Schulform*					
Grundschule (Referenz)	–	–	–	–	–
Oberschule	0,16	0,31	1,17	0,64–2,16	n. s.
Gymnasium	−0,15	0,32	0,86	0,46–1,62	n. s.
Gesamtschule	−0,05	0,39	0,95	0,44–2,06	n. s.
Förderschule	0,22	0,49	1,25	0,48–3,27	n. s.
*Sozioökonomischer Status*					
Niedrig (Referenz)	–	–	–	–	–
Mittel	−0,55	0,13	0,58	0,45–0,74	***
Hoch	−0,86	0,19	0,42	0,29–0,62	***

*B* Regressionskoeffizient *B*, *KI* Konfidenzintervall, *OR* Odds Ratio, *SE* Standardfehler, *UCLA* University of California, Los Angeles

* = *p* < 0,05, *** = *p* < 0,001, *n.* *s.* = nicht signifikant

In den multivariaten Analysen zeigt sich weiterhin bei beiden Erhebungsinstrumenten ein signifikanter Zusammenhang zwischen den Prädiktoren (Geschlecht, Alter und sozioökonomischer Status) und der Kriteriumsvariable Einsamkeit. Anders als in den bivariaten Analysen ergeben sich in der logistischen Regression unter Einschluss aller Variablen keine signifikanten Disparitäten bezüglich der Schulform.

## Diskussion

### Zusammenfassung und Einordnung zentraler Ergebnisse

Einsamkeit ist ein weit verbreitetes Phänomen, das in Deutschland bei Kindern und Jugendlichen noch vergleichsweise wenig Beachtung findet. Ziel der vorliegenden Studie war es, die Verbreitung von Einsamkeit bei Kindern und Jugendlichen in einer repräsentativen Studie für das Land Brandenburg zu erfassen und Zusammenhänge mit soziodemographischen Aspekten zu analysieren. Dazu wurden Schüler*innen an allgemeinbildenden Schulen mittels zweier Indikatoren zu ihrer Einsamkeit befragt. Das Ergebnis zeigt, dass sich zwischen 10,8 % (UCLA-Skala) und 16,8 % (Einzelitem) der befragten Kinder und Jugendlichen einsam fühlen. Die Ergebnisse der beiden Erhebungsinstrumente korrelieren in der vorliegenden Studie hoch miteinander. Dies korrespondiert mit früheren HBSC-Studien [26].

Die Ergebnisse zur Verbreitung liegen im Bereich der Befunde einiger früherer nationaler [44] und internationaler [9, 26, 39–41] Studien mit Heranwachsenden, während weitere Erhebungen wiederum deutlich geringere Häufigkeiten ermittelten [10]. Dabei ist jedoch u. a. zu berücksichtigen, dass Studien eine Zunahme von Einsamkeit bei Kindern und Jugendlichen über die Zeit hinweg berichten [9, 10]. Beispielsweise kann es im Rahmen der Corona-Pandemie zu einem Anstieg von Einsamkeit gekommen sein [1]. Insgesamt ist daher ein Vergleich der Befunde zur Verbreitung aufgrund unterschiedlicher Messzeitpunkte, Länder und Erhebungsmethoden nur bedingt möglich.

Entgegen der Ergebnisse von Eccles et al. [26] liegt die Einsamkeitsprävalenz in der vorliegenden Studie bei der Messung mit Einzelitem höher als bei der UCLA-Skala. Eine mögliche Interpretation hierfür könnte sein, dass es durch die zunehmende mediale Thematisierung von Einsamkeit während der Corona-Pandemie für die Heranwachsenden einfacher war, diesen Begriff mit den persönlichen Empfindungen in Verbindung zu bringen, während die komplexeren Abfragen aus der UCLA-Skala schwerer zuzuordnen waren. Der Vergleich mit den Befunden von Eccles et al. ist jedoch dadurch eingeschränkt, dass andere Skalierungen der Antwortoptionen und Formulierungen der Fragen verwendet wurden.

Einsamkeit war bei Mädchen im Vergleich zu Jungen mehr als doppelt so häufig (Tab. 1). Eine höhere Ausprägung von Einsamkeit bei Mädchen ist konsistent mit früheren Studien [9, 10, 26–28]. Eine Stärke der aktuellen Studie ist, dass sowohl direkte als auch indirekte Messmethoden verwendet wurden. Somit kann als Argument für eine höhere Prävalenz bei Mädchen nicht ihre stärkere Bereitschaft, sich als einsam zu bezeichnen [26], angeführt werden. Eine mögliche Erklärung für die häufigere Einsamkeit bei Mädchen kann sich aus einer geschlechtsspezifischen Sozialisation ergeben, welche Mädchen eher ermutigt, sich emotional zu öffnen und zwischenmenschlichen Beziehungen eine größere Bedeutung beizumessen [27]. Bei der kleinen Gruppe der Befragten mit der Geschlechtszuschreibung „divers“ war die Einsamkeitsprävalenz noch stärker ausgeprägt. Dieser Befund steht im Einklang mit einer früheren Studie mit Studierenden [49]. Als eine mögliche Interpretation dafür lässt sich anmerken, dass Menschen mit nichtbinärer Geschlechtsidentität auch häufiger unter psychischen Beeinträchtigungen leiden [49] und diese wiederum mit Einsamkeit assoziiert sein können [25]. Weiterhin stellt die Auseinandersetzung mit der eigenen Geschlechtsidentität und den Sorgen vor Diskriminierung und Stigmatisierung eine große Herausforderung dar [49].

Die Geschlechtsunterschiede blieben auch nach multivariater Prüfung in bedeutsamem Maße erhalten und zeigen für Mädchen im Vergleich zu Jungen ein doppelt bis 3‑fach so hohes Risiko und für Heranwachsende mit der Geschlechtszuschreibung „divers“ ein 7‑fach erhöhtes Risiko, häufig Einsamkeit zu erleben (Tab. 2).

Die Wahrscheinlichkeit, als „einsam“ kategorisiert zu werden, war bei beiden Erhebungsinstrumenten für Befragte aus der 9. Klasse im Vergleich zur 5. Klasse höher. Gemäß der UCLA-Skala war weiterhin die Wahrscheinlichkeit, als „einsam“ zu gelten, in der 7. Klasse höher als in der 5. Klasse. Eine Erklärung für diese je nach Messinstrument unterschiedlichen Ergebnisse könnte sein, dass die UCLA-Skala differenzierter Erfahrungen erfasst, die über den Verlauf von der Kindheit zur Jugend im Zusammenhang mit Einsamkeit eine immer wichtigere Rolle spielen. Beispielsweise hat das Bedürfnis, in der Peergruppe integriert zu sein („Ich fühle mich ausgeschlossen“), oder der Wunsch nach engen und intimen Bindungen („Ich fühle mich niemandem mehr nah“) in der Jugend eine größere Bedeutung als in der Kindheit [14].

Ein Anstieg der Einsamkeit mit dem Alter steht im Kontrast zu älteren Erhebungen mit Heranwachsenden aus Dänemark [10] und konvergiert mit neueren Befunden aus Finnland [9] und England [28]. Eine These für den Anstieg der Einsamkeit mit zunehmendem Alter könnte sein, dass über den Verlauf der Kindheit und Jugend zusätzliche Quellen der Einsamkeit entstehen. Während in der Kindheit insbesondere fehlende Spielpartnerschaften und Viktimisierungen eine Ursache von Einsamkeit sein können, sind es in der späteren Kindheit möglicherweise zusätzlich fehlende enge Freundschafen [14]. Jugendliche lösen sich zudem schrittweise vom Elternhaus, reflektieren darüber, welche Bedeutung soziale Isolation und zwischenmenschliche Beziehungen in ihrem Leben haben [15], und entwickeln vermehrt den Wunsch, in der Peergruppe anerkannt zu sein und romantische Beziehungen einzugehen [14]. Folglich kann auch in bisher zufriedenstellenden Beziehungen, wie mit den Eltern oder in der Peergruppe, Einsamkeit empfunden werden [12].

Bezüglich der Schulform ergaben die deskriptiven Analysen ein heterogenes Bild (Tab. 1). Gemäß dem Einzelitem zeigten sich signifikante Unterschiede in der Verbreitung von Einsamkeit, während dies bei der UCLA-Skala nicht der Fall war. Bei den multivariaten Analysen zeigen sich keine signifikanten Zusammenhänge mehr mit der Einsamkeit. Es ist daher davon auszugehen, dass weniger die Schulform selbst, sondern eher konfundierende Aspekte bei den bivariaten Untersuchungen für den ermittelten Zusammenhang verantwortlich waren. Dabei ist insbesondere eine Konfundierung der Schulform mit dem Alter zu berücksichtigen. Ein weiterer konfundierender Aspekt könnte eine Verbindung zwischen dem sozioökonomischen Status und der Schulform sein. So zeigt sich, dass Einsamkeit bei Heranwachsenden mit geringem sozioökonomischen Status häufiger ist. Dies entspricht vorherigen Befunden mit Heranwachsenden [10, 28]. Eine mögliche Erklärung dafür kann sein, dass Menschen mit geringerem sozioökonomischen Status über weniger Ressourcen und somit auch geringere soziale Teilhabemöglichkeiten verfügen.

### Limitationen

Die vorliegende Studie leistet einen wichtigen Beitrag zur Untersuchung der Einsamkeit bei Kindern und Jugendlichen in Deutschland. Sie erfasst mit einer großen repräsentativen regionalen Stichprobe die Verbreitung von Einsamkeit anhand zweier etablierter Erhebungsinstrumente und liefert so Daten zur Schließung einer Forschungslücke. Eine Limitation ist die vergleichsweise geringe Rücklaufquote auf Schulebene und auf Ebene der Schüler*innen, was die Möglichkeit einer Verzerrung durch systematische Ausfälle erhöht. Eine weitere Limitation ist, dass die verwendete 4‑Item-Variante der UCLA-Skala nicht zuvor an deutschen Kindern und Jugendlichen validiert, sondern lediglich in einem Pretest auf Verständlichkeit geprüft wurde. Ferner handelt es sich um eine regionale Stichprobe aus einem Bundesland, sodass eine Generalisierung der Befunde auf Deutschland nur bedingt möglich ist.

## Fazit

Die Ergebnisse der vorliegenden Studie zeigen, dass Einsamkeit bei Kindern und Jugendlichen weit verbreitet ist, sie liefern erste Hinweise zu relevanten Zusammenhängen zwischen Einsamkeit und soziodemographischen Merkmalen und zur Ableitung von Handlungsbedarfen für die Praxis. Nicht jede Ausprägung von Einsamkeit ist problematisch [39]. Dennoch ist zu berücksichtigen, dass ein hohes Maß an Einsamkeit bei Kindern und Jugendlichen mit Beeinträchtigungen der physischen und psychischen Gesundheit einhergehen kann [26, 28]. Die Schule ist ein geeigneter Ort, um möglichst viele Kinder und Jugendliche zu erreichen, Einsamkeit als verbreitetes Phänomen zu thematisieren und somit mögliche Stigmatisierungen von Einsamkeitsgefühlen zu reduzieren. Weiterhin können hier soziale und emotionale Kompetenzen gestärkt werden, was sich in Metaanalysen bereits als wirksam bei der Reduzierung von Einsamkeit bei Jugendlichen erwiesen hat [50].

Zusammenfassend konnte unsere Studie zeigen, dass der Risikofaktor „Einsamkeit“ bei deutschen Kindern und Jugendlichen ein weit verbreitetes und relevantes Phänomen darstellt, das in der Forschung und der Gesundheitsförderung weiterer Aufmerksamkeit bedarf.
